# Quality evaluation of physical properties, antinutritional factors, and antioxidant activity of bread fortified with germinated horse gram (*Dolichus uniflorus*) flour

**DOI:** 10.1002/fsn3.342

**Published:** 2016-01-20

**Authors:** Karishma Moktan, Pravin Ojha

**Affiliations:** ^1^ Department of Food TechnologyNational College of Food Science and TechnologyKathmanduNepal; ^2^ Food Research DivisionNepal Agricultural Research CouncilLalitpurNepal

**Keywords:** Antinutritional factors, antioxidant properties, bread, horse gram, polyphenol content

## Abstract

Horse gram was germinated at 90% RH at 25°C for 72 h after 24 h soaking and then grinded to pass through 150 *μ*m mesh size screens. The germination of horse gram result in increased protein, fiber, total polyphenol content, and antioxidant activity of horse gram flour whereas fat, ash, carbohydrate, iron, calcium, tannin, phytate, and oxalate were reduced due to germination. Bread was prepared by the incorporation of (2%, 4%, 6%, and 8%) germinated horse gram flour (GHF) by a straight dough method. The loaf volume and specific volume decreased with an increased use of percentage of GHF. The sensory evaluation revealed that the incorporation of GHF up to 6% was acceptable. The protein (% db), fiber (% db), ash (% db), iron (mg/100 g), calcium (mg/100 g), tannin (mg/g), phytate (mg/g), oxalate (mg/g), total polyphenol content (GAE/g), and antioxidant activity (DPPH % inhibition) was found to be 9.08 ± 0.01, 1.23 ± 0.15, 1.36 ± 0.11, 4.07 ± 0.03, 128 ± 0.26, 2.06 ± 0.15, 2.46 ± 0.15, 0.7 ± 0.1, 12.44 ± 0.40, and 31.13 ± 0.25, respectively, in 6% GHF incorporated bread. The research concludes that 6% GHF incorporation in bread enhance the polyphenol content and antioxidant properties.

## Introduction

A change in eating habit and increased population has increased bread consumption in developing countries (Siebel 2011). Wheat flour (WF), the vital ingredient for bread making has low protein content as well as lower protein quality due to deficiency in essential amino acid such as lysine and threonine (Jideani and Onwubali 2009; Young 2014). The use of composite flour for improving protein quality of bread and making functional bread has been increasing (Ndife et al. 2011; Oluwalana et al. 2012; Dooshima et al. 2014). Horse gram (*Dolichus uniflorus*) commonly known as *Gahat* is a traditional unexploited tropical grain legume, a cheapest source of protein and a good source of calcium, iron, and molybdenum, however, it is deficient in methionine and tryptophan as other legumes (Kadam and Salunkhe 1985; Bhokre et al. 2012). Horse gram is hard legumes grown in warmer areas in hills in marginal land with low fertility and is used as mainly for fodder purpose (Khadka 1987; Bhokre et al. 2012; Durga 2012). Sreerama et al. (2012) report higher polyphenol content and antioxidant activity in horse gram flour than cowpea and chickpea flour. Antioxidant property and enzyme activity modulation by phenolic content promote human health (Stanley and Aguilera 1985; McDougall and Stewart 2005). The use of horse gram flour in composite flour is restricted due to its antinutritional factors (Sreerama et al. 2012). Antinutritional factors not only hindered mineral absorption, protein digestibility, but also associated with “hard‐to‐cook phenomenon of legumes (Stanley and Aguilera 1985a; Amalraj and Pius 2015). Many antinutritional factors such as trypsin inhibitor, hemagglutinin activities, phytates, and tannin present in horse gram can be reduced by dehusking, germination, cooking, and roasting (Borade et al. 1984; Kadam and Salunkhe 1985; Bhokre et al. 2012). In vitro protein digestibility of horse gram was increased after germination (Ismail et al. 2003).

This research attempts to see the effect of partial replacement of WF by germinated horse gram flour (GHF) on sensory, chemical, and antioxidant activity of bread.

## Materials and Methods

### Preparation of bread

Horse gram collected from *Asan*, Kathmandu was cleaned thoroughly and soaked in water for 24 h at 25 ± 2°C in stainless steel utensils. It was then germinated in RH Chamber at the 25°C, 90% RH for 72 h as per the method of Ojha et al. 2014. The germinated horse gram seeds was dried in cabinet dryer (50°C for 6 h) and then grinded to a mesh size of 150 *μ*m.

Bread produced was formulated in the ratio of WF:GHF, respectively, in the ration of 100:0, 98:2, 96:4, 94:6, and 92:8 as shown in Tables 1 and 2.

**Table 1:** Formulation of bread with germinated horse gram flour (GHF)

Code	Wheat flour	GHF
A	100	0
B	98	2
C	96	4
D	94	6
E	92	8

**Table 2:** Ingredient used during bread making

Ingredient	Amount
Wheat flour + horse gram flour (g)	100
Sugar (g)	20
Salt (g)	0.4
Butter (g)	4
Water	65 mL
Yeast (g)	2

The bread was made by straight dough method as described by Kent and Evers (2004), which includes; mixing of raw material (according to formulation), propagation of yeast, addition of water and propagated yeast, kneading, first proofing, second proofing, molding, baking, and finally packaging.

### Analysis of raw material and bread

Proximate composition, iron content, calcium content, tanin, oxalate, and phytate were determined according to Ranganna (2008). Total polyphenol content was estimated by Folin–Ciocalteau Colorimetry as per Adom and Liu 2002;. Total Antioxidant activity was estimated by DPPH colorimetry method (Brand‐Williams et al. 1995). Bread characteristics were evaluated by measuring the loaf weight, loaf volume, and specific loaf volume using the rapeseed displacement method (Giami et al. 2004). The prepared bread was analyzed for sensory value using 9 points Hedonic Rating Test (9 = like extremely, 1 = dislike extremely) as per Ranganna (2008) for general appearance, crumb color, texture, flavor, and overall acceptability. Data obtained were analyzed by statistical program known as Genstat release 7.22, Discovery edition, 2004, developed by VSN International Ltd and t‐test by Microsoft Office Excel, 2007. Sample means were compared by LSD method at 95% level of significance.

## Results and Discussion

### Effect of germination on chemical and antioxidant activity of horse gram flour

The germination of horse gram resulted in an increase in protein, fiber, polyphenol, and antioxidant activity of horse gram flour, whereas fat, ash, carbohydrate, iron, calcium, tannin, phytate, and oxalate decreased due to the germination (Table 3).

**Table 3:** Chemical analysis of ungerminated (UHF) and germinated horse gram flour (GHF)

Parameters	UHF	GHF
Moisture (%)	12.2 ± 0.1^a^	12.36 ± 0.057^b^
Crude protein (%)	20.90 ± 0.11^a^	22.73 ± 0.13^b^
Crude fat (%)	0.81 ± 0.09^a^	0.53 ± 0.05^b^
Crude fiber (%)	5.23 ± 0.15^a^	5.83 ± 0.08^b^
Ash (%)	3.83 ± 0.10^a^	2.75 ± 0.04^b^
Carbohydrate (%)	69.21 ± 0.38^a^	68.16 ± 0.44^b^
Iron (mg/100 g)	7.03 ± 0.05^a^	6.4 ± 0.05^b^
Calcium (mg/100 g)	290 ± 0.01^a^	213.33 ± 1.14^b^
Tannin (mg/g)	16.18 ± 0.06^a^	10.28 ± 0.02^b^
Phytate (mg/g)	10.23.2 ± 0.15^a^	5.76 ± 0.15^b^
Oxalate (mg/g)	3.18 ± 0.01^a^	1.73 ± 0.05^b^
Polyphenol (mg GAE/g)	46.53 ± 1.13^a^	52.33 ± 0.57^b^
Antioxidant activity (DPPH% inhibition**)**	52.56 ± 0.75^a^	60.76 ± 0.64^b^

Data are mean value of triplicate determination ± standard deviation on dry basis except moisture.

Values followed by same superscript in the same column are not significantly different (*P* ≤ 0.05).

An increase in protein content might be due to biosynthesis of protein as a result of germination (Sattar et al. 1989; Bau et al. 1997; Nonogaki et al. 2010). The decrease in fat content might be due to use of fatty acid for energy for the germination (Bau et al. 1997; Hahm et al. 2008).

Crude fiber content of HF and GHF were found to be 5.23 ± 0.15% and 5.83 ± 0.08%, respectively. T‐test showed that germination had significant (*P* < 0.05) effect on the crude fiber content of horse gram. The increase in dietary fiber was reported to be mostly due to changes in the polysaccharides found in the cell wall such as cellulose, glucose, and mannose, suggesting that the changes were due to the synthesis of structural carbohydrates of cell such as cellulose and hemicellulose (Blessing and Gregory 2010; Rumiyati et al. 2012). During the germination, the ash content, calcium, and iron decreases due to leaching out during soaking (Saharan et al. 2001; Lestienne et al. 2005; Rusydi et al. 2011). The reduction in carbohydrate content was due to use of carbohydrate as source of energy for the embryonic growth (Vidal‐Valverde et al. 2002). Germination followed by soaking was reported to reduce antinutritional factor (Akande and Fabiyi 2010). During sprouting, phytic acid, a phosphate reserve degrades due to the action of phytase which is utilized by growing seedling (Mamudu et al. 2005). Polyphenolase activity during germination causes loss of tannins in grains during germination (Reddy et al. 1985). The reduction of oxalate may be due to germination (Khokhar and Apenten 2003). Increase in polyphenol and antioxidant activity was found after germination, which was also revealed by Tarzi et al. (2012).

### Physical characteristics of bread prepared from different composition of WF and GHF

Increase in percentage of GHF decreased the loaf volume and specific volume of bread, but increased the loaf weight (Table 4). Specific loaf volume was found to be greater than that of composite bread prepared by Dooshima et al. (2014); Ndife et al. (2011) and less compared to findings of Onuegbu et al. 2013; Oluwalana et al. 2012;. Loaf volume and specific volume decrease might be due to decrease in gluten network in dough and less ability of the dough to rise, due to weaker cell structure (Chavan and Kadam 1993; Abdelghafor et al. 2011). Increase in loaf weight may be due to increased absorption of water as revealed by Rao and Hemamalini (1991).

**Table 4:** Physical characteristics of bread prepared from different composition of wheat flour and germinated horse gram flour

Parameters	A	B	C	D	E
Loaf volume (cm^3^)	210.64 ± 0.33^a^	206.58 ± 0.16^b^	203.91 ± 0.28^b^	200.52 ± 0.37^b^	180.51 ± 0.32^c^
Loaf weight (g)	71.70 ± 0.25^a^	75.45 ± 0.72^b^	78.70 ± 0.63^b^	80.40 ± 0.43^b^	87.41 ± 0.48^c^
Specific volume (cm^3^/g)	2.93 ± 0.09^a^	2.73 ± 0.02^b^	2.59 ± 0.02^b^	2.49 ± 0.03^b^	2.06 ± 0.06^c^

Data are expressed as mean ± standard deviation (SD).

Values followed by same superscript in the same column are not significantly different (*P* ≤ 0.05).

### Sensory evaluation

**Figure 1:**
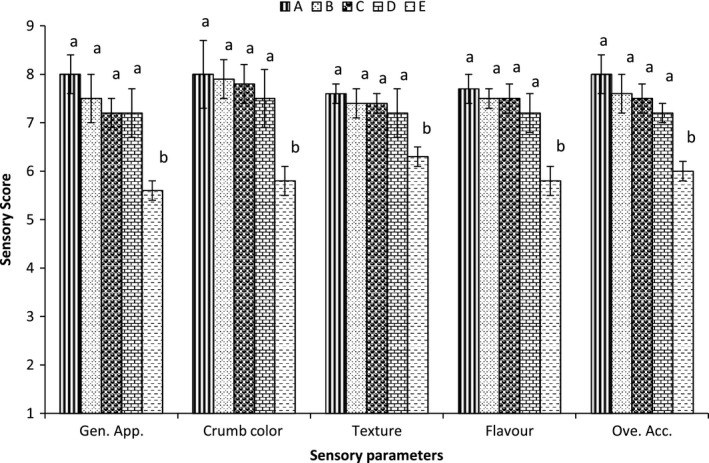
Effect of incorporation of gram flour on sensory score of bread. *Bar with same letter were not significantly different.

The results obtained showed that there were no significant differences in sensory score of all the sensory parameters up to 6% incorporation of GHF, however, 8% incorporation of GHF significantly reduced the sensory score of all the sensory parameters (Fig. 1). Bhokre et al. (2012) has also reported decrease in sensory acceptability with increase in percentage of GHF in bun.

Appearance of bread is an important sensory attribute for acceptability of bread (Hoseney 1994) Reduced sensory acceptability of composite flour bread for color with increase in percentage of other flour has been reported, which may be associated with increase in fiber (Singh et al. 2000; Hu et al. 2007; Akhtar et al. 2008; Serrem et al. 2011).

Increase in fiber due to wheat bran substitution in bread results in hard texture bread (Eimam et al. 2008). The texture of bread is affected by composition of bread, baking condition, and amount of water absorbed during mixing (Gomez et al. 2003). The decreased acceptability in terms of flavor can be linked with beany flavor (Okyoe and Okaka 2009). The overall acceptability may be affected by all the above sensory parameters.

### Chemical Analysis of the 6% GHF bread and comparison with control bread (WF bread)

On the basis of sensory evaluation, bread with 6% GHF was selected for further evaluation and compared with control bread (WF bread) for chemical properties (Table 5).

**Table 5:** Chemical analysis of wheat flour (WF) bread (control bread) and 6% germinated horse gram flour (GHF) bread (best product)

Parameters	Control bread	6% GHF bread
Crude protein (%db)	9.5 ± 0.43^a^	9.8 ± 0.01^a^
Crude fat (%db)	3.26 ± 0.22^a^	3.74 ± 0.25^a^
Crude fiber (%db)	0.37 ± 0.12^a^	1.23 ± 0.15^b^
Ash (%db)	0.03 ± 0.01^a^	1.36 ± 0.11^b^
Iron (mg/100 g)	1.52 ± 0.12^a^	4.07 ± 0.03^b^
Calcium (mg/100 g)	13.10 ± 0.95^a^	128 ± 0.26^b^
Tannin (mg/g)	0.47 ± 0.01^a^	2.06 ± 0.15^b^
Phytate (mg/g)	1.53 ± 0.05^a^	2.46 ± 0.15^b^
Oxalate (mg/g)	0.31 ± 0.01^a^	0.7 ± 0.1^b^
Total polyphenol (mg GAE/g)	0.13 ± 0.01^a^	12.44 ± 0.40^b^
Antioxidant activity (DPPH% inhibition)	4.27 ± 0.01^a^	31.13 ± 0.25^b^

Data are expressed as mean ± standard deviation (SD).

Values followed by same superscript in the same column for different products are not significantly different (*P* ≤ 0.05).

There is no increase in protein and fat significantly with incorporation of 6% GHF, however, fiber, ash, calcium, iron, tannin, phytate, oxalate, polyphenol, and antioxidant activity increased significantly. The increase in proximate and mineral content was due to high amount of these components in GHF compared to WF (Al‐Saleh and Brennan 2012; Marimuthu and Krishnamoorthi 2013).

The oxalate and phytate were lower than that reported by Malomo et al. (2011), however, tannin was found to be in higher side. Ojinnaka and Agubolum (2013) reported higher amount of tannin and phytate in cookies prepared from cashew nut. Phytate, oxalate, and tannin not only interferes with mineral absorption, but also impair protein digestibility (Raghubanshi et al. 2001; Gupta et al. 2006). Malomo et al. (2011) reported that 80 mg/g of these antinutritional factors are detrimental to health, so the values are considerable lower.

The total phenolic content was greater than that of bread prepared by substituting 50% of WF by mung bean, soybean, and mango kernel powder (Menon et al. 2014), and prepared by partly substitution of WF by lupin flour (Villarino et al. 2014), however, less than that of bread prepared by incorporating green tea and buckwheat (Bhattarai et al. 2012).

The increase in antioxidant in composite flour bread was also revealed by various authors (Bhattarai et al. 2012; Villarino et al. 2014; Irakli et al. 2015). Ingestion of food rich in antioxidant activity is associated with improvement of health status (Wang et al. 1997b).

## Conclusions

The germination of horse gram resulted in an increase in protein, fiber, total polyphenol content, and antioxidant activity of horse gram flour, whereas antinutritional factor such as tannin, phytate, and oxalate decreased. Bread prepared with incorporation up to 6% GHF was found to be acceptable in terms of sensory score. Total polyphenol and antioxidant activity along with mineral like calcium and iron content was increased significantly for 6% GHF incorporated bread.

## Conflict of Interest

None declared.
